# Supporting menstrual health in homeless services: provider-informed strategies for multilevel change

**DOI:** 10.1186/s12913-025-12932-1

**Published:** 2025-05-30

**Authors:** Emma Schnolis, Sofia Hrubiak, Anukriti Arora, Kinzie Gamaleldin, Rebecca Martinez, Risa Cromer, Yumary Ruiz, Natalia M. Rodriguez, Andrea L. DeMaria

**Affiliations:** 1 Department of Public Health, College of Health and Human Sciences, Purdue University, 812 West State Street, West Lafayette, IN 47907 USA; 2 School of Health Sciences, Purdue University, West Lafayette, IN USA; 3 Department of Chemistry, Purdue University, West Lafayette, IN USA; 4 Department of Anthropology, Purdue University, West Lafayette, IN USA

**Keywords:** Homelessness, Menstruation, Social service providers, Healthcare providers, Health equity, Social-ecological model

## Abstract

**Background:**

People experiencing homelessness (PEH) face heightened barriers to menstrual health, including limited access to products, hygiene facilities, and consistent care. Social service providers (SSPs) and healthcare providers (HCPs) are critical in supporting PEH but often work within resource-constrained systems. This study explores SSP and HCP perspectives on the menstrual health needs of PEH in a rural-serving community in the U.S., using the Social-Ecological Model (SEM) to identify multilevel barriers and opportunities for intervention.

**Methods:**

We conducted semi-structured interviews with 12 SSPs and HCPs in Tippecanoe County, Indiana. Transcripts were analyzed using thematic analysis to identify key challenges and recommendations related to menstrual health support for PEH.

**Results:**

Our analysis revealed complex challenges providers faced in supporting the menstrual health of PEH, including emotional burden, resource scarcity, and limited organizational guidance. They described how stigma, provider discomfort, and systemic gaps in training and infrastructure hindered effective care. Providers also shared that menstrual health was often deprioritized due to competing health needs and structural barriers such as lack of housing, transportation, and product access. Despite these challenges, providers offered actionable recommendations to improve menstrual health support through education, policy change, and more equitable organizational practices.

**Conclusions:**

This study highlights the multi-level barriers providers face when supporting the menstrual health needs of PEH. Our findings show that meaningful change requires coordinated efforts across all levels of the SEM. Actionable strategies include provider training, improved intake processes, expanded access to menstrual products and hygiene resources, and policy reforms to address housing and insurance gaps. These insights can inform training programs, shelter protocols, and advocacy efforts to promote menstrual health equity and provider sustainability.

**Supplementary Information:**

The online version contains supplementary material available at 10.1186/s12913-025-12932-1.

## Introduction

In 2022, it was estimated that 18 out of every 10,000 people in the United States were experiencing homelessness, an overall increase from 2020 [1]. Homelessness represents an intersection of structural inequities, where issues of poverty, race, gender, and access to healthcare converge, resulting in barriers to managing menstruation and securing necessary period products. Limited financial resources and lack of access to hygiene facilities create substantial obstacles to menstrual management. While many shelters offer products for free, access is constrained by proximity, available space, and resources [2– 4]. For people experiencing homelessness (PEH) without access to shelters, managing menstruation often involves improvising with inadequate materials or going without products entirely. A recent qualitative evidence synthesis found that some individuals forgo food, steal products, or use unsafe materials like rags and layered toilet paper to manage menstruation, practices that highlight the compounding effects of period poverty on an already vulnerable population [5]. This is echoed in recent research from Australia, which categorizes menstrual product insecurity into typologies and highlights similar coping mechanisms, including improvisation and prolonged product use, particularly among PEH, disability, and other forms of marginalization [6].

Period poverty has increased significantly during the past decade, as access to period products has become more limited in various areas [7], and maintaining adequate menstrual hygiene has become more challenging, further perpetuating health disparities among this population. Economic hardships make it difficult for PEH to obtain essential resources like medication, tampons, pads, and laundry services [8–13] necessary for myriad menstrual experiences. Instead of widespread initiatives, community organizations have taken individual actions to raise awareness about menstruation and well-being. Shelters and their residents have faced additional difficulties due to facility closures, inconsistent product donations, and disruptions caused by events such as the COVID-19 Pandemic [4]. Limited access to products and uncertainty about availability have prompted service providers to create distribution plans and initiate discussions about menstrual health with minimal external guidance [14]. This situation has created significant interpersonal challenges for both PEH and providers as they navigate the stigma often associated with menstruation, which can contribute to feelings of shame and discomfort during interactions. This study foregrounds how social service providers (SSP) and healthcare providers (HCP) navigate the challenges of providing menstrual health care for PEH in the context of period poverty and other systemic barriers.

HCPs and SSPs that serve the homeless community deliver care in an already challenging and complex healthcare system while trying to navigate the unique, multifaceted causes of homelessness, pervasive systemic barriers, and bias towards this vulnerable population that limit the access PEH has to healthcare. The patient-provider relationship is fundamental to providing high-quality healthcare. For menstruators, a spectrum of obstacles has manifested, including restricted period product distribution, closure of public facilities, and increased communication barriers [3], subsequently making interactions between providers and menstruators more complex. While some shelters and programs provide better access to primary care for PEH, many PEH patients still face challenges with transportation, financial, or time-sensitive resources to access these providers and their care [15]. Instead, PEH often resort to not receiving care or receiving care through the utilization of emergency departments [16]. This reliance on emergency room care strains the hospital system and its workers and prevents PEH from forming lasting, stable relationships with HCPs [12].

SSPs and HCPs who support this population often work in resource-limited environments, facing high caseloads, staff shortages, and organizational constraints shaped by funding inconsistencies across agencies [17]. These challenges are compounded when addressing menstruation-related needs, which may fall outside providers’ formal training and are sometimes perceived as uncomfortable or stigmatized topics. Providers have described the emotional strain associated with distributing products, navigating gender dynamics, and attempting to meet the hygiene needs of PEH without adequate resources or guidance [16, 17]. These challenges suggest a need to better understand how providers perceive and respond to menstrual health in the context of homelessness.

### Study purpose

This study explores how SSPs and HCPs perceive and navigate the menstrual health needs of PEH in Tippecanoe County, Indiana, applying the Social-Ecological Model (SEM) [18–20]. The SEM framework considers how individual, interpersonal, organizational, community, and policy-level factors influence health outcomes. In this study, the SEM helps situate the menstrual health needs of PEH within the broader systems that shape provider experiences and resource access [19].

Rather than directly capturing PEH’s lived experiences, we focus on the insights and experiences of those working to support this population. By centering provider perspectives, we examine the systemic and organizational barriers they encounter, the emotional and logistical challenges they face, and the strategies they employ to deliver menstrual health support. This study builds upon prior work in a small urban area that serves surrounding rural communities. It fills a gap in the existing literature, primarily focusing on larger urban centers like St. Louis and New York City [2, 3, 21]. Our findings and research offer critical insights into how public health systems and local organizations can better address menstrual health, offering actionable insights for policy, organizational improvement, and broader discourse on menstrual health equity for vulnerable populations.

## Methods

As part of a more extensive study, our research team conducted interviews with HCPs (i.e., registered nurses and EMTs) and SSPs (i.e., case managers and community health workers). Interviews were conducted with 12 participants from multiple organizations in Tippecanoe County, Indiana, to comprehensively understand their unique perspectives and challenges. We used a combination of purposive and convenience sampling to recruit participants with direct experience providing services to PEH. We aimed to reach thematic saturation rather than a predefined sample size. Saturation was assessed through continuous, iterative transcript review during data collection and coding. When no new codes or themes emerged in the final interviews, we determined that saturation had been reached. This study focused on the various experiences and viewpoints surrounding menstruation, policy, and health, as seen from the first-hand accounts of providers who served the homeless community.

### Study procedures

SSPs and HCPs were recruited through multiple methods, including direct outreach via public email and phone records and through preexisting personal connections with providers serving the homeless community. The recruitment phase was initiated in June 2021 and occurred until August 2022. To be eligible for participation in the study, all SSPs and HCPs needed to meet the following criteria: they had been employed as HCPs or SSPs, delivered services to PEH, and operated in Tippecanoe County. While purposive sampling aimed to capture a range of roles, including case managers, nurses, and EMTs, we encountered limitations in recruiting OB-GYNs and emergency room physicians, which may have constrained the range of perspectives represented. We acknowledge this as a potential study limitation and have elaborated on this in our Discussion section.

After expressing their willingness to participate, the providers completed an electronic consent form, provided verbal consent during the interview, and offered demographic information. The interviews followed a semi-structured protocol, with question prompts derived from a predetermined question list and adjusted to follow the flow of discussion (refer to Table 1 for example questions; the full interview guide has been provided as a supplemental file). Interviews were conducted in person (*n* = 3) or virtually (Zoom; *n* = 9), depending on participant preference and availability. All interviews followed the same semi-structured guide, ensuring consistency across formats. Following the interviews, SSPs and HCPs received electronic gift cards as compensation for their time.

**Table 1:** Interview questions

Primary Question	Probing Questions
How would you describe your area of expertise?	• How long have you been working in this field? • Can you tell me a little bit about your role/job at your organization? • What does a typical day for you look like in your role as a nurse, clinician, case manager, or housing specialist? • What has helped you the most to work in your field? (I.e. personal attribute, training, advice from a colleague or mentor) • What are some of the key things you do to support people experiencing homelessness?
How would you describe the community you work in?	• What are some positive attributes of the community you work in? Why? • What are some of the challenges of working in this community? Why? • How do you think this community view or define their health? • How does this community view health care? What is the purpose or reason to seek it out? • How would you describe the level of trust between you and the community you work in? • Do you think the people who come here other than staff would identify similar positive attributes and challenges? Why or why not?
In your role/organization, how and when do issues with menstruation come up?	• What does your organization offer for menstrual health for people experiencing homelessness? • What type of health education have you received for your job? Did this include menstrual health training? • What type of education or information related to menstruation is provided to people who come to your organization? What about other related information/education, such as women’s health or sexual health? How is this disseminated? • What menstrual products are available? How do people access these? [if none, why are products not available? ] • How do the available products meet or not meet people’s needs?
What spaces are available in your organization for menstruators to use to manage their menstruation? Such as toilets, showers, sinks, etc.	• How would you describe the condition of these spaces? • Where else do you think people experiencing homeless go to manage their menstruation? Hygiene? Access menstrual products?
Would you say your colleagues are generally more comfortable or uncomfortable talking about menstruation?	• What makes your colleagues most comfortable with this topic? Most uncomfortable? • What can be done to normalize this topic among your colleagues?
Thinking about menstruation among people experiencing homelessness, from your perspective, what is that experience like for them?	• Has anyone ever shared with you their experiences? What did you learn from those experiences? • What do you think are some of the challenges menstruators experiencing homelessness face when it comes to their menstrual health? • How do the challenges surrounding menstrual health affect other aspects of their health? • How do the challenges surrounding homelessness affect other aspects of their health? • Have you noticed or are you aware of any differences between newly vs. longer-term/chronic homeless individuals in terms of menstruation management?
Since the start of COVID-19, what challenges have you noticed about how people experiencing homelessness deal with menstruation and menstrual health?	• How have available supplies or spaces to change menstrual products changed? • What have you/your organization done to address challenges? • What are the largest challenges this population has faced?
What types of products do you believe should be offered to menstruators experiencing homelessness?	• Where should they be offered? • Who should pay for the products? Be responsible for overseeing the supply/demand? • How should the menstruation products offered differ from what is typically available? • Who should make the decisions about what products are available?
In what ways can service providers, like yourself, more effectively deal with the menstruation needs of people experiencing homelessness?	• How can the city more effectively support your clients with this need? [e.g. supplies, public toilets, disposal] • What would you recommend to policymakers? • If a training program was built to educate service providers on this topic, how do you think this should be delivered (e.g., online, in person, individually, group)? Should this be required? What would be helpful to you and your colleagues?

Otter.ai, a secure transcription software, was used to record and transcribe all interviews. Transcripts were reviewed for accuracy by multiple research team members. We then conducted a team-based thematic analysis guided by Braun and Clarke’s six-phase framework [22–24]. Coding was completed independently by multiple team members and reviewed collaboratively using NVivo 12 to support transparency and consistency. We developed a shared codebook, used thematic maps to refine and organize themes, and maintained an audit trail and analytic memos to document the evolving interpretation of the data. For further details on our methods, please refer to [20].

### Research team

Our research team included principal investigators with disciplinary expertise in public health, anthropology, and reproductive health and graduate and undergraduate students trained in qualitative research methods. Several team members had prior experience working with PEH and existing relationships with local service organizations, which helped facilitate trust and rapport during recruitment and interviews. At the same time, we recognize that our identities as researchers affiliated with a large academic institution, and in most cases not having lived experience of homelessness, may have shaped how participants responded to our questions and how we interpreted the data. We approached the study with an explicit commitment to reflexivity, regularly discussing how our perspectives, training, and social positions informed the research process. Throughout the project, we aimed to be mindful of these dynamics, using team debriefings, collaborative coding, and triangulation to help mitigate bias and support a rigorous, grounded analysis. Our awareness of these dynamics helped guide decisions about language used in interviews, interpretation of participant statements, and reflexive thinking throughout the research process.

## Results

Participant quotes are noted below to illustrate our findings. Quotes are followed by the interviewee’s professional title (i.e., [Healthcare Provider (HCP)], [Social Service Provider (SSP)]). Participant characteristics are described in Table 2. Four themes resulted from our analyses: strain and resource limitations among providers servicing PEH, menstrual health education and awareness for PEH, challenges in access to essential services, and provider recommendations for reform. These themes collectively highlighted the emotional difficulties that HCPs and SSPs face, the urgent need for menstrual health education, the obstacles hindering access to essential services for PEH, and the providers’ insights to improve an overburdened system. A complete listing of themes, subthemes, and representative participant quotes can be found in Table 3.

**Table 2:** Participant characteristics

	*N* = 12
**Age (years)**	37.25 ± 12.83
**Gender Identity**	
Woman (Cisgender or Transgender)	10 (83.33%)
Man (Cisgender or Transgender)	2 (16.66%)
**Sexual Orientation**	
Heterosexual or Straight	6 (50%)
Gay or Lesbian	3 (25%)
Bisexual	2 (16.66%)
Unsure	1 (8.33%)
**Identify as Transgender**	
No	12 (100%)
**Ethnicity**	
Hispanic or Latina/x	2 (16.66%)
Not Hispanic or Latina/x	10 (83.33%)
Prefer Not to Answer or did not respond	2 (12.50%)
**Race**	
White or Caucasian	9 (75%)
More than one race	2 (12.50%)
Asian or Asian American	1 (8.33%)
**Identify as a Person of Color**	
No	11 (91.66%)
Unsure	1 (8.33%)
**English as First Language**	
English Native Speaker	12 (100%)
**Political Views**	
Very Liberal	5 (41.66%)
Somewhat Liberal	5 (41.66%)
Somewhat Conservative	1 (8.33%)
Prefer not to answer or did not respond	1 (8.33%)
**Household Income**	
Comfortable	9 (75%)
Just enough to make ends meet	2 (12.50%)
Prefer not to answer or did not respond	1 (8.33%)

**Table 3:** Results summary table (Themes, Sub-themes, and representative Quotes)

Theme	Sub-Theme	Representative Quote
**Challenges** **(for SSP and HCP with PEH in relation to Menstruation Experience)**	Menstruation Awareness	“I worked in a place with a lot of poverty, and a lot of women didn’t have access to women’s healthcare. So, I had a lot of people who were heavy bleeding, they would call an ambulance because they didn’t know what’s going on. A lot of pregnancy calls, stuff like that, because they didn’t know what to do because they’d never been informed.” (HCP, Certified Recovery Specialist and EMT). “a lot of these women, not even specifically women, don’t ever get pap smears because… their primary care’s not going to tell them that they need one every year” (HCP, Certified Recovery Specialist and EMT).
**Challenges** **(for SSP and HCP with PEH in relation to Menstruation Experience)**	Organizational Processes	“… it’s just one of those things that if it comes up in conversation, then we’ll educate them…,” (HCP, EMT and Registered Nurse) “I think as we do the health intake, we need to redo our form to make sure we’re capturing that or at least ask everybody [to] make sure that you’re asking about menstruation cycles.” (HCP, Registered Nurse and Nurse Educator).
**Challanges (for SSP and HCP with PEH in relation to Menstruation Experience)**	Gender Dynamics	“They [PEH] have to ask staff at the desk for products. That’s unfortunate because some of our staff are male and some of our staff are judgy, and some of our staff are probably hoarders, you know, like you can have one, well I don’t want to come back in, you know, three hours…” (SSP, Chief Development Officer). “A lot of the case managers here are female…All but one of the community health workers here are female. And so I think they would feel more comfortable talking to them. But personally, nobody’s ever chatted with me about it. So, I think that’s just a matter of like a level of comfort.” (HCP, Registered Nurse and Nurse Educator)
**Challanges** **(for SSP and HCP with PEH in relation to Menstruation Experience)**	Job Turnover	“There are definitely quite a few [PEH] that I feel like I’ve built a trust with and that have approached me with topics, you know, that are really personal to them, but they just want to talk to me about them” (SSP, Guest Services) “I will admit it is a challenging field, I have had plenty of days where you have a guest who is angry at you for policies… we have had guests get in verbal arguments, physical arguments, we’ve had guests who have experienced seizures and medical emergencies…and seeing all the hardships of that can be stressful for mainly our guests but also, for the staff as well.” (SSP, Guest Services) “made the clients [PEH] suffer too” (SSP, Senior Director of Domestic Violence Intervention and Prevention).
**Challanges** **(for SSP and HCP with PEH in relation to Menstruation Experience)**	Strain and Resource Limitations	“It’s rough sometimes, really takes a toll on, you know like, my mental health and on my coworkers’ mental health. Just because there’s a lot of people with a lot of issues that you can’t get immediate resolution for. A lot of the people that we work with, it takes sometimes weeks to months to even get one issue resolved (HCP, Certified Recovery Specialist and EMT).
**Resources (SSP/ HCP perspectives on resources that impact Menstruation Experience for PEH)**	Housing	‘Well, it would be great if we had housing, and then they could be in their own space and get their own products and just deal with their lives, like everyone else has the opportunity to do… But short of getting them housing to deal with their periods, having an adequate supply of the products they are comfortable using is crucial’ (SSP, Chief Development Officer).
**Resources (SSP/ HCP perspectives on resources that impact Menstruation Experience for PEH)**	Clothes and Laundry	“We have had issues in the past with a shortage of smaller sizes in women’s underwear… and so those sizes like five and sixes ran out a little bit faster… if they, you know, had something happen to a pair of underwear, and we did have a washer and dryer, but you know, period events and underwear, if it’s bad enough, you did not really want to keep that same pair of underwear forever, so I could see that being an issue” (SSP, Guest Services). “And menstruation could cause issues with your clothing, especially if you were not expecting your period to start… or if they stained their clothing, you know that was the only clothing they had” (SSP, Guest Services).
**Resources (SSP/ HCP perspectives on resources that impact Menstruation Experience for PEH)**	Transportation	“most people walked if they didn’t have a car or they took the bus” (SSP, Day Center Director) “They [PEH] can’t walk to the store to buy them [menstrual hygiene products] or other things… if they have open infections, if they have infection in their legs that will negatively affect their overall health, so which then relates the menstrual health” (HPC, Recovery Specialist and EMT).
**Resources (SSP/ HCP perspectives on resources that impact Menstruation Experience for PEH)**	Menstruation Products/ Items	“SNAP doesn’t pay for hygiene products, it doesn’t pay for toiletries, toilet paper, that kind of stuff. So that’s a limitation” (SSP, Mental Health and Addictions Counselor) “There’s no government assistance for that type of thing” (HCP, Certified Recovery Specialist and EMT) “The front desk is not allowed to hand out any pain relief or medication because we’ve had issues with people abusing it in the past, and we’re just, we’re not allowed to hand them out anymore” (SSP, Guest Services)
**Resources (SSP/ HCP perspectives on resources that impact Menstruation Experience for PEH)**	Other (Healthcare Accessibility, Population Safety)	“We do health assessments on people…we try and determine if they need help, getting a new provider, getting insurance, what kind of health problems they do have… that we need to try and help them improve” (SSP, Case Manager). “I think there’s just all kinds of risks related to their sexual health and safety there so, you know, and if you have a risk factor in that category that just opens up your risk factors in other categories” (SSP, Chief Development Officer).

### Provider strain in resource-limited, emotionally demanding settings

#### Emotional burden

SSPs and HCPs who serve PEH experience emotional strain. This emotional strain was frequently due to feelings of inability to help clients, as providers faced countless systemic barriers and policies that hindered comprehensive care. One HCP described:

It’s rough sometimes, really takes a toll on, you know like, my mental health and on my coworkers’ mental health. Just because there’s a lot of people with a lot of issues that you can’t get immediate resolution for. A lot of the people that we work with, it takes sometimes weeks to months to even get one issue resolved (HCP, Certified Recovery Specialist and EMT).

Providers agreed that limited access to resources and restrictive organizational policies caused difficulty when providing services to PEH in various areas. One frequently discussed example was the requirement of PEH to rely on SSPs and HCPs to obtain period products. While PEH reported feelings of vulnerability and embarrassment when asking providers for period products, providers also experienced difficult emotions as the distributors. This issue was illustrated when one provider described when a young girl “had to go ask him [a male employee] for products, and I’m sure that was horribly, just embarrassing and humiliating for her to have to do” (SSP, Chief Development Officer). Many providers in our study also reported that, in their facilities, menstrual products were distributed directly by staff rather than made available in public restrooms, often as a way to manage limited supplies. This restriction, stemming from limited organizational capacity and insufficient menstrual product availability, added to the emotional strain on providers, many of whom expressed a desire to offer more support to PEH but were constrained by the resources at their disposal. 

#### Job turnover and staff instability

Job turnover was frequently mentioned by SSPs and HCPs, and inconsistency in staffing “made the clients [PEH] suffer too” (SSP, Senior Director of Domestic Violence Intervention and Prevention). Many PEH noted that they appreciated having repeated interactions with providers [16], and high job turnover rates inhibited the development of trust between providers and PEH. As one SSP described, “There are definitely quite a few [PEH] that I feel like I’ve built a trust with and that have approached me with topics, you know, that are really personal to them, but they just want to talk to me about them” (SSP, Guest Services). High job turnover can lead to inconsistent staffing schedules, introducing additional challenges to the complex relationship between PEH and providers. This inconsistency in staffing forces current providers to exert extra effort and employ innovative approaches to acquire the knowledge required to address the needs of PEH effectively, consequently intensifying the emotional strain on the providers.

Providers also experience job-related stress when PEH have negative healthcare experiences. In their roles, providers have had difficulty helping PEH “develop trust again in the healthcare system to where they’ll actually go see a provider” (HCP, Registered Nurse and Nurse Educator). Amidst the demanding landscape of serving PEH, providers navigated through numerous challenges, from addressing guest frustrations with policies to managing altercations among guests, as highlighted by one SSP’s account,

I will admit it is a challenging field, I have had plenty of days where you have a guest who is angry at you for policies… we have had guests get in verbal arguments, physical arguments, we’ve had guests who have experienced seizures and medical emergencies…and seeing all the hardships of that can be stressful for mainly our guests but also, for the staff as well. (SSP, Guest Services)

Providers described how the emotional challenges of supporting PEH, particularly around sensitive issues like menstruation, added to the stress of an already demanding work environment. These findings reflect interpersonal-level dynamics within the SEM, where provider-client relationships are strained by systemic limitations and emotional labor, highlighting how broader structural constraints shape frontline experiences.

### Menstrual health education and awareness for PEH

#### Awareness

SSPs’ perspectives highlighted a significant need for increased education and awareness among staff regarding menstrual health for PEH. One SSP, who previously served as a first responder, shared:

I worked in a place with a lot of poverty, and a lot of women didn’t have access to women’s healthcare. So, I had a lot of people who were heavy bleeding, they would call an ambulance because they didn’t know what’s going on. A lot of pregnancy calls, stuff like that, because they didn’t know what to do because they’d never been informed. (HCP, Certified Recovery Specialist and EMT).

The lack of information was partly due to the neglect of conversations between providers and their clients, which resulted in decreased reception of essential healthcare services. One provider noted, “a lot of these women, not even specifically women, don’t ever get pap smears because… their primary care’s not going to tell them that they need one every year” (HCP, Certified Recovery Specialist and EMT). However, the sheer amount of information that HCPs were responsible for discussing during appointments with PEH, coupled with frequent injuries and illnesses exacerbated by homelessness, led to menstrual health becoming a low priority. Another social service provider elaborated on the systemic barriers that limit menstruation-related education and treatment, saying, “they’ll [PEH] go to the ER for a lot of things, but I mean the ER isn’t really an area where you go for regular gynecological evaluations or exams and stuff like that so. So, they just kind of put it off” (SSP, Mental Health and Addictions Counselor). This marginalized focus on menstrual health was not indicative of provider neglect but rather a consequence of the compounding health issues facing PEH and the systemic barriers including insurance coverage and primary care reimbursement that limited proper menstrual health education.

#### Gender dynamics

Lack of knowledge about menstrual health contributed to perceived gender dynamics between male-identifying SSPs and menstruating PEH. SSPs in our study described male-identifying providers as often uninformed about essential aspects of menstruation. Combined with the stigma surrounding menstruation, their lack of knowledge created issues of perceived comfort with the topic, as one provider noted, “We’ve got one male case manager he may be, like, touchy about that [menstruation]” (SSP, Case Manager). This made it difficult for male providers to allocate menstruation resources and healthcare services effectively and challenged PEH who may have needed advice or products. As one provider described:

They [PEH] have to ask staff at the desk for products. That’s unfortunate because some of our staff are male and some of our staff are judgy, and some of our staff are probably hoarders, you know, like you can have one, well I don’t want to come back in, you know, three hours… (SSP, Chief Development Officer).

Limited awareness of menstrual health affected male staffers, leaving them uninformed and perpetuating stigma surrounding menstruation.

Gender dynamics between male-identifying providers and PEH exacerbated the sense of vulnerability PEH experienced when broaching the topic of menstruation, particularly with male providers. One male-identifying provider acknowledged that discussions about menstruation rarely arose in his daily interactions, emphasizing that PEH often preferred addressing such matters with female providers. He explained, “A lot of the case managers here are female…All but one of the community health workers here are female. And so I think they would feel more comfortable talking to them. But personally, nobody’s ever chatted with me about it. So, I think that’s just a matter of like a level of comfort.” (HCP, Registered Nurse and Nurse Educator). The interactions above illustrate a degree of discomfort experienced by both male providers and PEH around the education and conversations of menstruation. More cognizant efforts by all providers to open conversations around menstruation amongst each other and with PEH can alleviate this discomfort. Additionally, educating male providers can offset the unintentional perpetuation of the stigma, in turn restoring trust and vulnerability in the provider-PEH relationship. Lastly, these efforts may reduce the emotional burden of female providers by distributing labor across all providers.

### Processes

Providers emphasized the need for improved education among staff members and raised concerns about the absence of menstruation-related questions in their intake forms. They highlighted that failing to gather this information hindered their ability to comprehensively assess menstruators’ needs, leaving PEH without essential health information. As one provider suggested, “I think as we do the health intake, we need to redo our form to make sure we’re capturing that or at least ask everybody [to] make sure that you’re asking about menstruation cycles.” (HCP, Registered Nurse and Nurse Educator). Additionally, another noted: “… it’s just one of those things that if it comes up in conversation, then we’ll educate them…,” (HCP, EMT and Registered Nurse) indicating that discussions about menstrual health did not occur unless initiated by PEH themselves. Not proactively eliciting menstrual health information can contribute to insufficient communication and unmet needs.

This theme illustrates organizational-level barriers in the SEM, such as inadequate training, gendered staffing imbalances, and missing intake procedures, which influence how menstrual health is addressed within care settings.

### Challenges in access to menstruation-related resources for PEH

#### Period products

Community organizations in the Lafayette area contributed to menstrual health by supplying PEH with period products like pads and tampons. For many PEH, obtaining free products through community shelters was their only option, as they could not afford to pay for products, and governmental assistance did not cover period products. One provider explained, “SNAP doesn’t pay for hygiene products, it doesn’t pay for toiletries, toilet paper, that kind of stuff. So that’s a limitation” (SSP, Mental Health and Addictions Counselor), while another provider concurred, saying, “There’s no government assistance for that type of thing” (HCP, Certified Recovery Specialist and EMT). Like clothing, the shelter’s supply of pads and tampons was based on “what’s donated,” limiting the style, type, and size of the products they received and distributed. One provider noted that some PEH were occasionally dissatisfied or unhappy with the products they were given, suggesting that "…It would be maybe a good idea at some point to, you know, ask the population what they want” (SSP, Guest Services).

Menstruation can come with symptoms of pain and discomfort, which created a challenging situation for staff who worked in shelters and interacted with PEH. Shelter staff were limited in the resources they could provide to manage pain, and one SSP described, " The front desk is not allowed to hand out any pain relief or medication because we’ve had issues with people abusing it in the past, and we’re just, we’re not allowed to hand them out anymore” (SSP, Guest Services). Another provider mentioned that the shelter used to provide “heating pads,” but now, all they had to offer were “hand warmers” likely due to availability (SSP, Mental Health and Addictions Counselor). 

#### Clothes and laundry

Providers emphasized that limited access to clean clothing and laundry services created significant challenges for menstruating PEH, which not only affected hygiene and dignity but also placed added strain on staff trying to meet these needs with insufficient resources. Many relied on donations and shelter resources for clothing and undergarments, which led menstruating PEH to reuse or wear soiled clothing. To address this, one community organization offered free laundry services, alleviating the financial burden for PEH. One provider highlighted the importance of these services, noting:

Not having access to that [money] would very much inhibit them being able to launder their clothes. Because if they did not have money, they were not going to be able to go to a laundromat, there’s like nowhere else you gonna do it” (SSP, Case Manager).

Another provider explained, “And menstruation could cause issues with your clothing, especially if you were not expecting your period to start… or if they stained their clothing, you know that was the only clothing they had” (SSP, Guest Services). Access to laundry services was crucial for providers to offer, especially for menstruating PEH, to ensure PEH could maintain sanitary practices, uphold their hygiene, and prevent staining from menstrual fluids.

However, organizational policies and service demand still posed challenges. High demand and limited operating hours of facilities created barriers for PEH and difficulties for SSPs. When these services were not readily available, PEH needed to rely on shelter donations for new clothing, which could be challenging due to size limitations in donations received. As one provider noted:

We have had issues in the past with a shortage of smaller sizes in women’s underwear… and so those sizes like five and sixes ran out a little bit faster… if they, you know, had something happen to a pair of underwear, and we did have a washer and dryer, but you know, period events and underwear, if it’s bad enough, you did not really want to keep that same pair of underwear forever, so I could see that being an issue (SSP, Guest Services).

Several providers noted that limited access to laundry services contributed to hygiene challenges for menstruating PEH, which in turn created additional strain on staff trying to meet these needs. Furthermore, the destruction of undergarments due to the lack of laundry services posed difficulties for SSPs due to the frequent lack of variability of supplies and sizing.

#### Housing

In transitional housing and homeless shelters, staff offered a broad range of services to the PEH community, primarily focusing on securing housing. One SSP’s perspective on this process was shared: “We work really hard with community partners to help people, not just get into housing but also get into services they need to maintain that housing in hopes to not, you know, be homeless again later” (SSP, Day Center Director). Access to housing significantly impacted the physical and mental health of PEH, allowing them to maintain personal hygiene and menstrual health, which could be challenging without consistent living accommodations. One provider emphasized the importance of access to housing for menstrual health:

Well, it would be great if we had housing, and then they could be in their own space and get their own products and just deal with their lives, like everyone else has the opportunity to do… But short of getting them housing to deal with their periods, having an adequate supply of the products they are comfortable using is crucial (SSP, Chief Development Officer).

Overall, ensuring PEH are housed allowed for safe, hygienic, private space for menstruators to maintain their menstrual health, improving their physical health.

#### Transportation

Providers frequently cited transportation as a major barrier affecting PEH’s ability to access menstrual health services and other forms of care. PEH typically lacked personal transportation and had to rely on public transit, friends, family, or walking to attend appointments and access shelter services. According to one provider, “most people walked if they didn’t have a car or they took the bus” (SSP, Day Center Director). Walking to appointments posed challenges for many PEH and risked decreasing appointment attendance by making healthcare less accessible. One provider explained that a lack of transportation affected menstruation management for some PEH through increased incidence of podiatric problems stating, “They [PEH] can’t walk to the store to buy them [menstrual hygiene products] or other things… if they have open infections, if they have infection in their legs that will negatively affect their overall health, so which then relates the menstrual health” (HPC, Recovery Specialist and EMT). The inconsistent ability of many PEHs to ambulate between stores, appointments, and shelters poses a significant obstacle to menstruation management.

Additionally, without regular and consistent healthcare access, menstruation persisted as an under-discussed topic due to myriad other illnesses and injuries that took precedence at healthcare appointments and shelter intake appointments. Furthermore, the broader need for transportation between shelters for meals, emergency accommodations, and personal hygiene facilities like bathrooms, showers, and laundry added additional complications for menstruators in terms of upholding menstrual hygiene. These challenges span the community and policy levels of the SEM, showing how housing instability, transportation, public infrastructure, and policy gaps (like lack of SNAP coverage for products) compound menstruation-related barriers for PEH.

### Provider recommendations for reform

#### Provider suggestions

Providers agreed that targeting structural and organizational issues was pertinent to alleviate the additional emotional burden from the demanding careers of HCPs and SSPs. As one SSP stated, “as far as trusting healthcare and different, you know, organizations, we can kind of break down those barriers with people by doing outreach” (SSP, Certified Peer Recovery Coach). Another HCP described, “sometimes there’s not enough resources available for the amount of population” (HCP, EMT and Registered Nurse). Increasing access to menstrual health resources for PEH can reduce the stress SSPs feel when distributing supplies. Some providers believed that increasing outreach and awareness around menstrual health could help build trust with clients and ease some of the emotional burden they experience in their daily roles.

Menstrual health is vital to overall well-being, yet discussions and education between providers and PEH remained lacking. To address this gap, one provider proposed essential questions for health assessments, suggesting “…asking those specific questions of are you having a regular period? Are…your periods like, are they heavy…” (HCP, EMT and Registered Nurse). When queried about steps to eliminate menstrual health stigma, one provider answered: “I think just education across the board, like their experiences and really killing the stigma.” (HCP, EMT and Registered Nurse). Reforms in the education, processes, and care approaches employed by providers are imperative for delivering effective care that empowers PEH and places menstrual health at the forefront of care plans. 

#### Additional challenges

Furthermore, challenges in healthcare delivery and existing comorbidities add further obstacles for providers to navigate when serving PEH. Variables such as employment status, housing status, and health status make it difficult for PEH to obtain insurance, and thus to receive healthcare. One provider described the process of combating this, stating, “We do health assessments on people…we try and determine if they need help, getting a new provider, getting insurance, what kind of health problems they do have… that we need to try and help them improve” (SSP, Case Manager). Financial restrictions due to insurance status made facility options scarce, and PEH often resorted to an emergency department or general facility. Ultimately, medical services became limited and generalized without insured PEH, limiting providers’ ability to comprehensively treat or manage patient health.

These issues were often exacerbated in menstrual health with relevance for the broader sexual and reproductive health contexts, as preventative care was often derived from primary care, and primary care may have been unattainable. As one provider described, “I think there’s just all kinds of risks related to their sexual health and safety there so, you know, and if you have a risk factor in that category that just opens up your risk factors in other categories” (SSP, Chief Development Officer). Many health issues continually worsen when not addressed by providers and ensuring that SSPs and HCPs monitor ongoing health issues can lead to improved menstrual health outcomes within PEH. Providers’ recommendations target multiple SEM levels (i.e., organizational (e.g., intake reform), community (e.g., expanded outreach), and policy (e.g., menstrual equity legislation)), indicating that effective solutions must operate across systems.

## Discussion

This study applied the SEM [19] to understand the multilevel factors shaping how HCPs and SSPs support the menstrual health needs of PEH. The SEM helps unpack the complex interplay of individual, interpersonal, organizational, community, and policy-level barriers impacting providers and PEH. Using this model, we identified layered intervention points that can improve care provision, reduce provider burden, and advance menstrual equity in homeless services. 

### Individual

 At the individual level, providers reported limited menstrual health training and, in some cases, discomfort discussing menstruation, particularly among male-identified or non-menstruating staff [2, 3]. These knowledge gaps often led to delayed or insufficient support for PEH. PEH also lacked essential reproductive health knowledge, often due to missed preventive care and low health literacy, further complicating their ability to advocate for their menstrual needs. Similar findings emerged from a study from Brazil that found that PEH were significantly more likely to use toilet paper or rags as menstrual products and to experience resulting skin irritation, infections, and emotional distress, emphasizing the urgent need for targeted menstrual health education and resources among vulnerable populations [25]. Structured education for providers and PEH is essential. Menstrual health should be part of onboarding for staff, especially for non-clinical and male-identifying providers. For PEH, menstrual health education during intake can promote informed care-seeking. 

### Interpersonal

Trust between providers and PEH is central to delivering effective, compassionate care. However, high job turnover, staffing shortages, and emotional burden disrupt these relationships and erode trust over time [1, 16]. Participants shared that forming consistent, trusting relationships was critical for discussing sensitive topics like menstruation, yet often difficult to maintain due to workforce instability and emotional fatigue. Given the emotional toll described by providers, implementing trauma-informed practices, reflective debriefings, and peer support mechanisms may mitigate these emotional burdens and help preserve interpersonal continuity. Addressing menstrual health must include interventions that support providers’ mental well-being to ensure sustainable, relational care for PEH. 

### Organizational

Within organizations, menstrual health was rarely prioritized, often due to outdated or insufficient intake forms, lack of policies regarding menstrual product distribution, or poor infrastructure for maintaining hygiene. Products were typically stored behind desks or distributed inconsistently, creating privacy concerns and contributing to stigma. Orsini et al. [5] similarly found that menstruation management was often contingent on irregular donations, product gatekeeping by staff (often male), and lack of private or hygienic facilities, further marginalizing menstruating individuals. Sometimes, providers felt forced to ration products or turn people away due to low supplies, creating tension and dissatisfaction among staff and clients [3, 7]. Organizations should prioritize structural changes that institutionalize menstrual equity, such as updating intake forms to include menstruation-related questions, placing products in private restrooms, and ensuring staff across all roles are prepared to address menstruation with sensitivity and accuracy. These changes can standardize care and reduce provider discomfort, while also promoting dignity for PEH. 

### Communal

Community organizations served as essential intermediaries, especially without robust public support. However, their efforts were constrained by inconsistent donations, variable funding, and limited physical resources. Providers reported ongoing challenges in meeting PEH’s needs for clothing, laundry services, and accessible hygiene facilities, all critical to menstrual management [4, 7]. Community-level interventions should include increased shelter funding and donation drives focused on menstrual and hygiene products. Cross-sector partnerships can also enhance resource-sharing and expand mobile or decentralized hygiene services. These improvements are particularly critical in rural-serving communities like the study setting, where services are dispersed, and transportation remains a barrier. 

### Policy

Systemic policy gaps continue to undermine menstrual equity. Federal benefits such as SNAP, WIC, and Medicaid do not cover period products, and many states, including Indiana, still apply sales tax to these items [2, 3, 7]. As a result, shelters and individuals must rely on informal support systems, often leading to product shortages and rationing. Providers also emphasized the need for better access to affordable housing and reliable transportation, which affect menstrual health management and continuity of care. Local and statewide policy reforms should focus on expanding access to menstrual products and providing direct funding to shelters for product procurement. Housing-first models and transportation subsidies for PEH can reduce instability and improve long-term health outcomes, including menstruation. As Cooke [26] explains, donation-dependent systems reflect deeper inequities, where menstrual hygiene is treated as a charitable offering rather than a guaranteed right. More sustainable solutions include direct government funding, policy mandates for free products in public facilities, and corporate accountability measures [6].

### Implications for health professionals and policymakers

Framing the findings through the SEM reveals a clear need for multilevel responses to support menstrual health equity for PEH. Health professionals can benefit from targeted training in menstrual health, trauma-informed care, and stigma reduction, especially for non-clinical staff and male-identified providers. Organizational leaders should implement standardized intake tools, product distribution policies, and emotional support mechanisms for staff to promote consistency in care and support provider well-being. Investments in affordable housing and transportation infrastructure are also critical to ensure PEH can manage their menstruation with dignity and access consistent, supportive care. By addressing these issues across SEM levels, we can create sustainable, dignity-centered solutions that benefit PEH and the providers serving them.

### Strengths and limitations

A strength of this study is its interdisciplinary research team and community-engaged approach, which enabled rich, candid provider insights into a frequently overlooked aspect of homelessness services. The study’s setting in a rural-serving, mid-sized community fills an essential gap in the literature, primarily focusing on large urban centers. However, several limitations must be noted. First, the sample lacked representation from OB-GYNs and emergency department physicians, which may have constrained the clinical perspectives captured. The demographic composition, primarily white, cisgender women, also limits the generalizability of cultural or gender-specific responsiveness findings. Finally, this study centers on provider perspectives; future research should include PEH voices directly to understand menstrual health experiences and fully co-develop responsive solutions.

## Conclusion

This study highlights the challenges HCPs and SSPs face in supporting the menstrual health of PEH. Using the SEM, we show how barriers exist at multiple levels: individual, interpersonal, organizational, community, and policy. Supporting providers through improved education on menstrual health, along with increasing access to housing, transportation, and period products for PEH, are all critical. Findings can directly inform training programs, shelter protocols, and policy reforms, helping ensure PEH receive menstrual health support with dignity, consistency, and compassion.

## Supplementary Information

Supplementary Material 1.

## Data Availability

Data will be made available in the Purdue University Research Repository (https://purr.purdue.edu/).
