# Supplementary material for: Supporting menstrual health in homeless services: provider-informed strategies for multilevel change
Source: BMC Health Serv Res. 2025 May 30;25:778. doi: 10.1186/s12913-025-12932-1 (PMC12123704; doi:10.1186/s12913-025-12932-1)
Supplement: Supplementary file 1 — Supplementary Material 1. [file 12913_2025_12932_MOESM1_ESM.docx]

# Service Providers of Menstruators Experiencing Homelessness: Interview Questions

*Updated 11.8.21*

**Understanding the Menstrual Health Needs of People Experiencing Homelessness during the COVID-19 Pandemic in Lafayette, Indiana**

PIs: Natalia Rodriguez, PhD, MPH; Department of Public Health, College of Health and Human Sciences; Purdue University; West Lafayette, IN; USA; natalia@purdue.edu; Andrea L. DeMaria, PhD, MS; Department of Public Health, College of Health and Human Sciences; Purdue University; West Lafayette, IN; USA; ademaria@purdue.edu; Yumary Ruiz, PhD, MPH: Department of Public Health, College of Health and Human Sciences; Purdue University; West Lafayette, IN; USA; ruiz46@purdue.edu; Risa Cromer, PhD: Department of Anthropology, College of Liberal Arts; Purdue University; West Lafayette, IN; USA; risacromer@purdue.edu

Welcome. I want to start by saying how thankful I am that you are here today to help me with this research project I am conducting with my research team about menstruation.

My name is ________________ and I will be conducting this research interview. I am part of an interdisciplinary team at Purdue University conducting research on community and health issues, including menstruation. [If note taker is present add in: “This is_______ and [she/he/they] will be taking notes throughout our meeting today so that I can spend my time concentrating on our conversation and your answers.]

First, I want to thank you for completing the consent form and participant survey. As a brief reminder, the goal of the study is to understand what healthcare and social service providers know, experience, perceive, and practice around menstrual management for people experiencing homelessness.

Your experiences are important to me. As the interviewer, I will ask you questions related to menstruation and community resources. As the participant, you are encouraged to share experiences you feel comfortable with, which will be kept completely anonymous. I want you to know that your opinions are very important and there are no right or wrong answers. You do not have to answer all questions, you can ask me to skip a question, and you can end the interview at any time. If you need clarification on any question, please do not hesitate to ask me to further explain.

As was indicated in the consent form, I will be audio recording today’s conversation because I do not want to miss any of your comments. But, your name will always remain confidential, so anything we discuss today will not be tied to your name in any final reports or publications and your healthcare provider will not know you participated or have access to anything you say in the interview.

My role today is to ask questions and listen. I will be moving the discussion from one question to the next. During our talk, I may sometimes have to interrupt you if I feel we are running out of time and there are several more questions to get to; please do not feel this has to do with what you are saying.

What questions do you have before we get started? [wait a few moments]

Ok, great! I will now begin the audio recording. From here on forth, both of our voices will be captured. Just to confirm, do I have your permission to audio record today’s interview? Yes, or No?

**The first question I am going to ask is about your professional training and current position. [spend about 5 minutes here]**

1. **How would you describe your area of expertise? [Following questions will be adjusted to their specific field/expertise]**

Probe: How long have you been working in this field?

Probe: Can you tell me a little bit about your role/job at your organization?

Probe: What does a typical day for you look like in your role as a nurse, clinician, case manager, or housing specialist?

Probe: What has helped you the most to work in your field? (I.e. personal attribute, training, advice from a colleague or mentor)

Probe: What are some of the key things you do to support people experiencing homelessness?

1. **How would you describe the community you work in?**

Probe: What are some positive attributes of the community you work in? Why?

Probe: What are some of the challenges of working in this community? Why?

Probe: How do you think this community view or define their health?

Probe: How does this community view health care? What is the purpose or reason to seek it out?

Probe: How would you describe the level of trust between you and the community you work in?

Probe: Do you think the people who come here other than staff would identify similar positive attributes and challenges? Why or why not?

**Next, I would like to ask about services offered for people who menstruate. [spend about 30 minutes here]**

1. **In your role/organization, how and when do issues with menstruation come up?**

Probe: What does your organization offer for menstrual health for people experiencing homelessness?

Probe: What type of health education have you received for your job? Did this include menstrual health training?

Probe: What type of education or information related to menstruation is provided to people who come to your organization? What about other related information/education, such as women’s health or sexual health? How is this disseminated?

Probe: What menstrual products are available? How do people access these? [if none, why are products not available?]

Probe: How do the available products meet or not meet people’s needs?

1. **What spaces are available in your organization for menstruators to use to manage their menstruation? Such as toilets, showers, sinks, etc.**

Probe: How would you describe the condition of these spaces?

Probe: Where else do you think people experiencing homeless go to manage their menstruation? Hygiene? Access menstrual products?

1. **Would you say your colleagues are generally more comfortable or uncomfortable talking about menstruation?**

Probe: What makes your colleagues most comfortable with this topic? Most uncomfortable?

Probe: What can be done to normalize this topic among your colleagues?

1. **Thinking about menstruation among people experiencing homelessness, from your perspective, what is that experience like for them?**

Probe: Has anyone ever shared with you their experiences? What did you learn from those experiences?

Probe: What do you think are some of the challenges menstruators experiencing homelessness face when it comes to their menstrual health?

Probe: How do the challenges surrounding menstrual health affect other aspects of their health?

Probe: How do the challenges surrounding homelessness affect other aspects of their health?

Probe: Have you noticed or are you aware of any differences between newly vs longer-term/chronic homeless individuals in terms of menstruation management?

1. **Since the start of COVID-19, what challenges have you noticed about how people experiencing homelessness deal with menstruation and menstrual health?**

Probe: How have available supplies or spaces to change menstrual products changed?

Probe: What have you/your organization done to address challenges?

Probe: What are the largest challenges this population has faced?

1. **What types of products do you believe should be offered to menstruators experiencing homelessness?**

Probe: Where should they be offered?

Probe: Who should pay for the products? Be responsible for overseeing the supply/demand?

Probe: How should the menstruation products offered differ from what is typically available?

Probe: Who should make the decisions about what products are available?

**Finally, I would like to talk about ways to improve our community and workforce related to menstruation. [spend about 5 minutes here]**

1. **In what ways can service providers, like yourself, more effectively deal with the menstruation needs of people experiencing homelessness?**

Probe: How can the city more effectively support your clients with this need? [e.g. supplies, public toilets, disposal]

Probe: What would you recommend to policymakers?

Probe: If a training program was built to educate service providers on this topic, how do you think this should be delivered (e.g., online, in person, individually, group)? Should this be required? What would be helpful to you and your colleagues?

**Conclusion**

1. **Is there something else that you would like to share with me about your experiences that you think is important that we have not discussed?**

Those are all the questions I have. I appreciate your time in speaking with me, and your openness and honesty in sharing your professional experience. Are there any final comments you would like to share with me before we conclude our interview?

Ok, I am now ending the audio recording. Anything you say from here on will not be captured.

Thank you for participating today. Your insights are valuable for the success of our research and we appreciate your time. **We will be emailing you your gift card. Please be on the lookout for it this week.**

As a reminder, the study investigator contact information is on your consent form. Please do not hesitate to reach out with any questions, or if you would like to be connected to the study website that will house preliminary results.

If you think of anyone who may be a good fit for this study or have interest in participating, please feel free to pass my contact information along and have them reach out to me about the study.

Thank you and have a great day!
